# Pharmacological and Molecular Effects of Platinum(II) Complexes Involving 7-Azaindole Derivatives

**DOI:** 10.1371/journal.pone.0090341

**Published:** 2014-03-06

**Authors:** Pavel Štarha, Jan Hošek, Ján Vančo, Zdeněk Dvořák, Pavel Suchý, Igor Popa, Gabriela Pražanová, Zdeněk Trávníček

**Affiliations:** 1 Department of Inorganic Chemistry, Regional Centre of Advanced Technologies and Materials, Faculty of Science, Palacký University, Olomouc, Czech Republic; 2 Department of Cell Biology and Genetics, Regional Centre of Advanced Technologies and Materials, Faculty of Science, Palacký University, Olomouc, Czech Republic; 3 Department of Human Pharmacology and Toxicology, Faculty of Pharmacy, University of Veterinary and Pharmaceutical Sciences Brno, Brno, Czech Republic; University of Quebect at Trois-Rivieres, Canada

## Abstract

The *in vitro* antitumour activity studies on a panel of human cancer cell lines (A549, HeLa, G-361, A2780, and A2780R) and the combined *in vivo* and *ex vivo* antitumour testing on the L1210 lymphocytic leukaemia model were performed on the *cis*-[PtCl_2_(*n*aza)_2_] complexes (**1**–**3**) involving the 7-azaindole derivatives (*n*aza). The platinum(II) complexes showed significantly higher *in vitro* cytotoxic effects on cell-based models, as compared with *cisplatin*, and showed the ability to avoid the acquired resistance of the A2780R cell line to *cisplatin*. The *in vivo* testing of the complexes (applied at the same dose as *cisplatin*) revealed their positive effect on the reduction of cancerous tissues volume, even if it is lower than that of *cisplatin*, however, they also showed less serious adverse effects on the healthy tissues and the health status of the treated mice. The results of *ex vivo* assays revealed that the complexes **1**–**3** were able to modulate the levels of active forms of caspases 3 and 8, and the transcription factor p53, and thus activate the intrinsic (mitochondrial) pathway of apoptosis. The pharmacological observations were supported by both the histological and immunohistochemical evaluation of isolated cancerous tissues. The applicability of the prepared complexes and their fate in biological systems, characterized by the hydrolytic stability and the thermodynamic aspects of the interactions with cysteine, reduced glutathione, and human serum albumin were studied by the mass spectrometry and isothermal titration calorimetric experiments.

## Introduction

*Cisplatin* is a simple platinum(II) coordination compound that is used world-wide for the treatment of various types of cancer [1], [2]. The discovery of its antitumour effect on human cancer cells in 1960s [3], [4] and its consequent approval by the Food and Drug Administration for the therapeutic use in 1978 represent an important milestone in the field of both bioinorganic and medicinal chemistry. Representing a relatively uncomplicated leading compound, hundreds and hundreds of platinum(II) complexes were prepared using diverse strategies how to modify the structure and biological action of *cisplatin*. Two basic approaches focused either on the substitution of the leaving groups, *i.e.* two chlorides (*e.g.* in *carboplatin* [5] or *nedaplatin* [6]), or on the substitution of two NH_3_ molecules within the *cisplatin* molecule by different *N*-donor ligands (*e.g.* in *oxaliplatin* [7] or *lobaplatin* [8]) represent the most promising ways leading towards clinically useful platinum(II) compounds. Nevertheless, none of these compounds avoided completely both of the main disadvantages related with the platinum-based drugs application, *i.e.* the resistance (acquired or intrinsic), and negative and dose-limiting side effects (nephrotoxicity, neurotoxicity, myelosuppression etc.) [1], [2], [9].

Since 1978, the development of new *cisplatin*-inspired bioactive complexes seemed many times as a lost cause, nevertheless the preparation of several novel highly-active compounds proved that there is still a room for the improvement of pharmacological properties of antitumour platinum complexes [10]. One of the possible future research directions was shown in the case of *picoplatin* [11]. A rational replacement of one NH_3_ molecule in the *cisplatin* molecule by one relatively simple heterocyclic *N*-donor ligand (2-methylpyridine) led to the lower interaction with sulphur-containing biomolecules (*e.g.* glutathione) resulting in lower inactivation of the substance and, as a consequence of this, in a notable ability to overcome resistance of various tumour types to the action of *cisplatin* and *oxaliplatin* [11]–[13]. Although *picoplatin* failed in the clinical trials on non-small-cell lung carcinoma due to the continued progression of the disease and showing several drawbacks (*e.g.* neutropenia, thrombocytopenia or vomiting), it is currently undergoing clinical trials as therapeutic for colorectal and prostate cancer [14].

**Figure 1:**
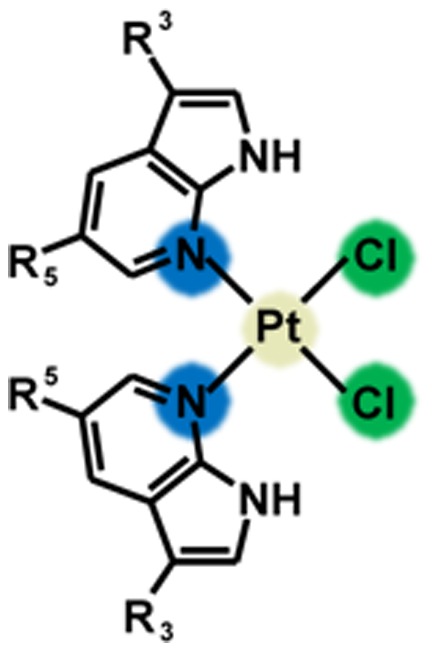
General structural formula of the studied platinum(II) complexes. R_3_ = Cl for **1**, I for **2** and H for **3**; R_5_ = H for **1**, H for **2** and Br for **3**.

Bearing this in mind, we aimed to find a simple, planar and well-coordinating *N*-donor heterocycle, whose incorporation into the *cisplatin* molecule, instead of one or both NH_3_ molecules, could bring in the similar effect on the antitumour properties as in the case of *picoplatin*. Thus, we chose 7-azaindole and its halogeno derivatives, which fulfil the mentioned requirements [15]–[17]. Moreover, the biological perspective of 7-azaindole moiety, as recently proved on various 7-azaindole derivatives reported as having notable biological properties, such as anticancer activity [18], inhibition of kinases (*e.g*. tropomyosin-related [19] or Abl and Src kinases [20]) or antiviral effect [21], has to be taken into account as well. Recently, we prepared and thoroughly characterized the platinum(II) dichlorido complexes involving 7-azaindole or its halogeno derivatives 3-chloro-7-azaindole (the complex **1** in this work), 3-iodo-7-azaindole (**2** in this work) and 5-bromo-7-azaindole (**3** in this work) (see Figure 1) and screened them for their *in vitro* antitumour activity against HOS osteosarcoma, MCF7 breast carcinoma and LNCaP prostate carcinoma human cancer cell lines with IC_50_ equalled 1.5–8.0 µM [22], [23]. In addition, the results of mechanistic studies confirming their analogous mechanism of action to *cisplatin* and significantly higher cell-uptake, intracellular transport and DNA platination, resulting in the higher *in vitro* effectiveness as compared with *cisplatin* were recently reported [23], [24].

Following the previous promising results of *in vitro* studies, we were determined to perform an advanced study of *in vitro* cytotoxicity on an extended panel of human cancer cell lines (A549, HeLa, G-361, A2780 and *cisplatin*-resistant A2780R), together with the *in vivo* and *ex vivo* studies on L1210 lymphocytic leukaemia model complemented by the histological and immunohistochemical investigation on the cancerous tissues and studies of expression of caspases 3 and 8, p53 and VEGF-A, *i.e.* the proteins associated with the tumour growth progression and induction of apoptosis. In this paper, we present the results of thorough biological testing, extended by the data regarding the applicability of the compounds and the stability of their solutions in water containing media.

## Materials and Methods

### Ethics Statement

This study was carried out in strict accordance with the recommendations in the Guide for the Care and Use of Laboratory Animals of the National Institute of Health. The protocol was approved by the Expert Committee on the Protection of Animals Against Cruelty at the University of Veterinary and Pharmaceutical Sciences Brno (Permit Number: 42/2011). To minimize the suffering of laboratory animals, the number of pharmacological interventions was limited to the necessary minimum and the animals were observed regularly for any signs of unnecessary suffering from the symptoms of the tumor progression. All animals showing at least one of the following symptoms - weight loss higher than 50% of the initial weight, symptoms of acute toxicity caused by the tested compounds, and excessive volume of the tumors hindering the mobility or social communication of the animals, had to be sacrificed immediately by cervical dislocation. However, no such cases occurred within the whole 21 days of pharmacological testing. The animal tissues for the *ex vivo* experiments were taken post mortem, immediately after all animals were sacrificed by the cervical dislocation.

### Chemicals and Biochemicals

K_2_[PtCl_4_], 3-chloro-7-azaindole (*3Cl*aza), 3-iodo-7-azaindole (*3I*aza), 5-bromo-7-azaindole (*5Br*aza) and solvents (acetone, methanol, ethanol, diethyl ether, *N,N′*-dimethylformamide, water) were purchased from the following commercial sources - Sigma–Sigma-Aldrich Co. (Praha, Czech Republic), Acros Organics Co. (Pardubice, Czech Republic) and Fisher-Scientific Co. (Pardubice, Czech Republic). The complexes *cis*-[PtCl_2_(*3Cl*aza)_2_] (**1**), *cis*-[PtCl_2_(*3I*aza)_2_] (**2**) and *cis*-[PtCl_2_(*5Br*aza)_2_] (**3**) (Figure 1) were synthesized by the reactions of water solution of K_2_[PtCl_4_] with two molar equivalents of *n*aza dissolved in ethanol, and characterized as reported previously [23].

The RPMI 1640 medium and penicillin-streptomycin mixture were purchased from Lonza (Verviers, Belgium). Phosphate-buffered saline (PBS), fetal bovine serum (FBS), phorbol myristate acetate (PMA), Auranofin (98%≤), erythrosin B, and *Escherichia coli* 0111:B4 lipopolysaccharide (LPS) were purchased from Sigma-Aldrich (Steinheim, Germany). Cell Proliferation Reagent WST-1 was obtained from Roche (Mannheim, Germany). Instant ELISA Kits (eBioscience, Vienna, Austria) were used to evaluate the production of TNFα and IL-1β.

### NMR Spectroscopy

^1^H, ^13^C and ^195^Pt NMR spectra and two dimensional correlation experiments (^1^H–^1^H gs-COSY, ^1^H–^13^C gs-HMQC, ^1^H–^13^C gs-HMBC; ^1^H–^15^N gs-HMBC; gs =  gradient selected, COSY =  correlation spectroscopy, HMQC =  heteronuclear multiple quantum coherence, HMBC =  heteronuclear multiple bond coherence) of the DMF-*d_7_* solutions were measured at 300 K on a Varian 400 device at 400.00 MHz (^1^H), 100.58 MHz (^13^C), 86.00 MHz (^195^Pt) and 40.53 MHz (^15^N). ^1^H and ^13^C spectra were adjusted against the signals of tetramethylsilane (Me_4_Si). ^195^Pt spectra were calibrated against potassium hexachloroplatinate (K_2_PtCl_6_) in D_2_O found at 0 ppm. ^1^H–^15^N gs-HMBC experiments were obtained at natural abundance and calibrated against the residual signals of DMF adjusted to 8.03 ppm (^1^H) and 104.7 ppm (^15^N). The splitting of proton resonances in the reported ^1^H spectra is defined as s =  singlet, d =  doublet, t =  triplet, br =  broad band, dd =  doublet of doublets, m =  multiplet.

Stability study: the DMF-*d_7_* solutions of **1**–**3** were studied by ^1^H NMR after 6 h, 24 h and 1 week and by all the above-mentioned NMR experiments after two weeks of standing at laboratory temperature, and by ^1^H NMR after 15 min and 1 h of heating at 100°C.

Hydrolysis study: the complexes **1** and **3** (*ca.* 20 mg) were dissolved in 0.5 ml of DMF-*d_7_* and 0.25 ml of distilled water were added. The mixture was heated to 50 or 100°C for 3 h and white solid formed. The product was removed and dissolved in DMF-*d_7_*. The purity of the hydrolyzed products was >95%.

### The Interaction and Stability Studies of 1 Evaluated by the ESI-MS

The 10 µM methanolic solution (final concentration) of the selected complex *cis*-[PtCl_2_(*3Cl*aza)_2_] (**1**) was mixed with the equivalent volume of L-cysteine (Cys) and reduced glutathione (GSH) dissolved in water in the physiological concentrations (the final concentration of 260 µM for Cys, and 6 µM for GSH, respectively) [25]. The mixture was sealed and kept at 25°C for a month. During this period (immediately after the preparation, 1 h, 12 h and 1 month after the preparation), the small amounts of the reacting mixture (usually 20 µL) were analyzed by the FIA-ESI-MS method in both the positive and negative ionization mode. The mobile phase composed of 90% methanol and 10% of 10 mM ammonium acetate was pumped at the rate of 0.2 mL/min by the quaternary pump of Dionex Ultimate 3000 HPLC System. The samples were injected (50 µL) into the flow of the mobile phase by the autosampler of the HPLC from the same make. The parameters of Thermo LCQ Fleet mass spectrometer were set as follows: API Source voltage 4.5 kV, sheath gas flow rate 40 (arb units), aux gas flow rate 20 (arb units), capillary temperature 275°C, RF lens voltage −2.91 V, lens0 −5.89 V, lens1 −8.89 V, gate lens −43.94 V, multipole1 offset −11.87 V, front lens −70.71 V. The similar FIA-ESI-MS experiment was arranged to test the stability of the selected complex **1** in a water/methanol solution (10 µM, 1∶1 v/v) over the same time period as mentioned above.

### ITC Experiments

All the isothermal titration calorimetry (ITC) measurements were performed at 30°C using a VP-ITC device (MicroCal Inc.). The studied solutions (2.5 µM HSA, 100.0 µM GSH, 100.0 µM Cys, 1.0 mM *cisplatin* and 1.0 mM **1**) in a water/DMF mixture (1∶1 v/v) were degassed prior to the titration. The experiments (titration of 100.0 µM Cys with 1.0 mM solution of **1**, titration of 100.0 µM GSH with 1.0 mM solution of **1**, titration of 2.5 µM HSA with 1.0 mM solution of **1**, titration of 100.0 µM Cys with 1.0 mM solution of *cisplatin*, titration of 100.0 µM GSH with 1.0 mM solution of *cisplatin* and titration of 2.5 µM HSA with 1.0 mM solution of *cisplatin*) were carried out uniformly: the biomolecule (HSA, GSH or Cys) was titrated with **1** (or *cisplatin*) by 25 injections of 10 µL each with interval between injections being 300 s. The blank experiments were performed using the same conditions without appropriate biomolecule in cell, where only the water/DMF (1∶1 v/v) mixture was poured. The blank experiment data were subtracted and the data were fitted (one-site, two-site or sequential binding model) using the MicroCal Origin software version 7.0.

### Cell Culture and *In Vitro* Cytotoxicity Testing

*In vitro* cytotoxic activity of **1**–**3** and the clinically used platinum-based drug *cisplatin* was determined by an MTT assay in Human Negroid Cervix Epitheloid Carcinoma (HeLa; ECACC No. 93021013), Human Caucasian Malignant Melanoma (G-361; ECACC No. 88030401), Human Ovarian Carcinoma (A2780; ECACC No. 93112519), Human Ovarian Carcinoma *Cisplatin*-resistant Cell Line (A2780R; ECACC No. 93112517) and Human Caucasian Lung Carcinoma (A549; ECACC No. 86012804) cancer cell lines purchased from European Collection of Cell Cultures (ECACC). The cells were cultured according to the ECACC instructions and they were maintained at 37°C and 5% CO_2_ in a humidified incubator. The human cancer cells were treated with **1**–**3** and *cisplatin* (applied up to 50 µM) for 24 h, using multi-well culture plates of 96 wells. In parallel, the cells were treated with vehicle (DMF; 0.1%, v/v) and Triton X-100 (1%, v/v) to assess the minimal (*i.e.* positive control) and maximal (*i.e.* negative control) cell damage, respectively. The MTT assay was measured spectrophotometrically at 540 nm (TECAN, Schoeller Instruments LLC).

The data were expressed as the percentage of viability, when 100% and 0% represent the treatments with DMF and Triton X-100, respectively. The cytotoxicity data from the cancer cell lines were acquired from three independent experiments (conducted in triplicate) using cells from different passages. The IC_50_ values were calculated from viability curves. The results are presented as arithmetic mean±SD.

### *In Vivo* Antitumour Activity

The testing of *in vivo* antitumour activity was carried out according to the previously published procedure using the female DBA/2 SPF mice as experimental animals [26]. In this specific case, the animals were housed in the Sealsafe NEXT – IVC Blue Line Housing System (Tecniplast, Italy) to ensure the best possible experimental conditions and eliminate the risk of possible inter-group cross-contamination. Due to the lack of toxicological data and in order to eliminate the excessive use of laboratory animals, the experimental setup, involving the use of the same dose for all the platinum(II) complexes (3 mg/kg) was used. The animals were weighed daily and observed several times a day for the signs of tumour progression, changes in behaviour, sudden death, and sacrificed if their body weight decreased below 50% of the starting weight or if other severe toxicological problems were seen. The experimental data, describing the survival of the experimental animals, were expressed as the percentage of mean survival time, %T/C defined as the ratio of the mean survival time of the treated animals (T) divided by the mean survival of the untreated control group (C). There were no deaths attributable to acute toxicity of the tested compounds.

### Histological and Histochemical Evaluations

The tissue samples, obtained by the dissection of the sacrificed animals were divided into two parts, the first one was kept below −80°C for further evaluation by the methods of molecular biology, and the second one was processed by the histological procedures as described previously [26]. The paraffin embedded preparations were stained by the standard hematoxylin and eosin staining. The immunohistochemical detection of the Caspases 3 and 8, tumour necrosis factor-alpha (TNF-α) and transcription factor p53 expression were achieved by the use of appropriate antibodies, selective for the mouse species, obtained from AbCam (Cambridge, UK). The quantification of the tissue expression of different proteins in the samples was semi-quantitative, based on the percentage of the areas containing the detected proteins in the view field of at least three different samples. The scale from 0 to 4 was used, where 0 =  protein was not detected, 1 =  up to 25%, 2 =  up to 50%, 3 =  up to 75% and 4 =  up to 100% of the area contains the detected protein within the view field. The median value from at least three observations was used to evaluate the expression of selected proteins and other histological observations.

### Protein Expression Analysis

Frozen tumour samples were homogenized by a bench blender IKA DI25 (IKA-Werke, Germany) in the presence of a lysis buffer [50 mM Tris-HCl pH 7.5, 1 mM EGTA, 1 mM EDTA, 1 mM sodium orthovanadate, 50 mM sodium fluoride, 5 mM sodium pyrophosphate, 270 mM sucrose, 0.5% (v/v) Triton X-100]. Subsequently, the blended samples were centrifuged at 7.000 g at 4°C for 15 min. The supernatants were collected and the protein concentration was measured by the Brandford's method using the Brandford reagent (Amresco, USA) according to a manufacturer's manual. The measured samples were stored at −80°C for the following experiments.

The concentration of VEGF-A was evaluated by ELISA using VEGF-A Platinum ELISA (eBioscience, Austria). The amount of produced VEGF-A was normalized according to the total protein concentration.

The expression of caspase 3, caspase 8 and p53 were evaluated by Westernblot and immunodetection. Lysates were mixed with a denaturing loading dye [250 mM Tris-HCl pH 6.8, 5% (v/v) β-mercaptoethanol, 10% (w/v) SDS, 30% (v/v) glycerol, 0.04% (w/v) bromophenol blue], heated for 5 min at 70°C and loaded into a 12% polyacrylamide denaturing gel. The final protein amount was 50 µg per well. After protein separation in the gel, they were transferred on the supported nitrocellulose membrane 0.2 µm (Bio-Rad, USA) and the proteins were visualized by Ponceau S dye (Sigma-Aldrich, USA) for loading control. Then, the membrane was blocked by 5% (w/v) BSA at TBST [10 mM Tris-HCl pH 7.5, 150 mM NaCl, 0.1% (v/v) Tween-20]. The following primary and secondary antibodies [conjugated with horseradish peroxidase (HRP)] were used for immunodetection: caspase 3 (eBioscience, USA) in the dilution of 1∶1000, caspase 8 (Abcam, UK) in the dilution of 1∶1000 and p53 (Abcam, UK) in the dilution of 1∶1000, goat anti-mouse antibody in the dilution of 1∶3000 (Sigma-Aldrich, USA), goat anti-rabbit antibody in the dilution of 1∶3000 (Sigma-Aldich, USA). HRP was detected by Amplified Opti-4CN kit (Bio-Rad, USA). One sample from the control group was loaded into all gels to avoid inter-gel variability. The amount of the proteins was determined by densitometric analysis using AlphaEasy FC 4.0.0 software (Alpha Innotech, USA) and was correlated according to the control sample.

### Zymography

Samples were prepared by the same way as for immunodetection. Zymography of MMPs was performed in the gelatin impregnated gel as was described previously [27]. For the analysis, 30 µg of proteins obtained from lysates were loaded in a gel. The intensity of the digested regions was calculated by AlphaEasy FC 4.0.0 software (Alpha Innotech, USA) for densitometric analysis. The results were normalized according to 1% (v/v) fetal bovine serum (FBS) (Sigma-Aldrich, USA), which was used in each gel as a standard control.

### Statistical Evaluation

The significance of the differences between the results was assessed by the ANOVA analysis, followed by Tukey's post-hoc test for multiple comparisons, with *p*<0.05 considered to be significant (QC Expert 3.2, Statistical software, TriloByte Ltd.).

## Results and Discussion

### Synthesis and General Properties

**Figure 2:**
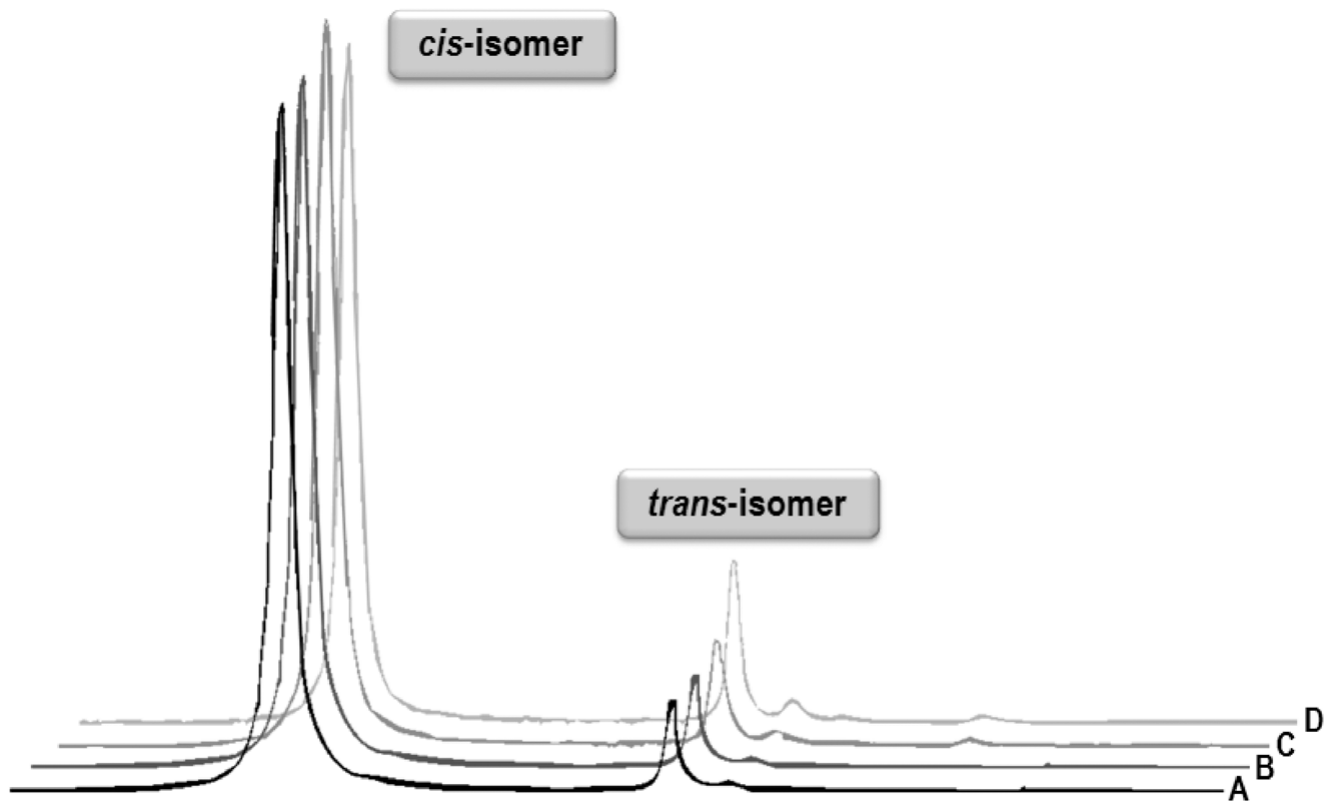
Part of the ^1^H NMR spectra of *cis*-[PtCl_2_(*5Br*Haza)_2_] (3). The spectra show the N1–H signal of *cis*- (left) and *trans*-isomers (right) as observed at different times and temperatures: (A) fresh solution at laboratory temperature; (B) after two weeks at laboratory temperature; (C) after 15 min at 100°C; (D) after 1 h at 100°C.

The studied complexes *cis*-[PtCl_2_(*3Cl*aza)_2_] (**1**), *cis*-[PtCl_2_(*3I*aza)_2_] (**2**) and *cis*-[PtCl_2_(*5Br*aza)_2_] (**3**) (Figure 1) were prepared by the one-step reactions of 7-azaindole halogeno-derivatives (*n*aza) with K_2_[PtCl_4_] at 50°C as described previously [23], yielding the products of high chemical purity as demonstrated by ^1^H, ^13^C, ^15^N and ^195^Pt NMR data, with isomeric purity >95% (based on ^1^H NMR data; see Figure 2A).

### Stability Studies

The NMR spectra of the studied complexes in DMF-*d_7_* did not show any difference after two weeks at laboratory temperature and even after 15 min at 100°C. We observed a slight increase of *trans*-isomer portion (see the data in File S1 labelled as **1t**, **2t**, and **3t**, which correspond to the *trans*-isomers of the complexes **1**, **2**, and **3**, respectively; the NMR data for the corresponding *cis*-isomers were already published in our previous work [23]) after 1 h at 100°C (Figure 2). In addition, the hydrolytic stabilities of the complexes **1** and **3** were investigated by means of ^1^H NMR spectroscopy in the mixture of DMF-*d_7_*/H_2_O (2∶1 v/v). We did not detect any ^1^H NMR spectra changes even after one week at laboratory temperature. However, the set of new signals was detected at the spectra of the complexes heated in the mentioned DMF-*d_7_*/H_2_O mixture to 50°C or 100°C (see the data in File S1 labelled as **1 h** and **3 h** for the hydrolytic products of the complexes **1**, and **3**, respectively). These changes are most probably connected with the hydrolysis of the studied complexes in the mentioned water-containing system, since the chemical shifts of the new signals do not correspond to either *cis*- (NMR data given in [23] or *trans*-isomer (the NMR data are given in File S1) of the studied complexes or to the signals of free *n*aza molecules. Probably, these signals may be associated with the formation of the hydrolytic products/species, such as [Pt(*n*aza)_2_(OH)_2_] or [Pt(*n*aza)_2_(H_2_O)_2_]^2+^.

**Figure 3:**
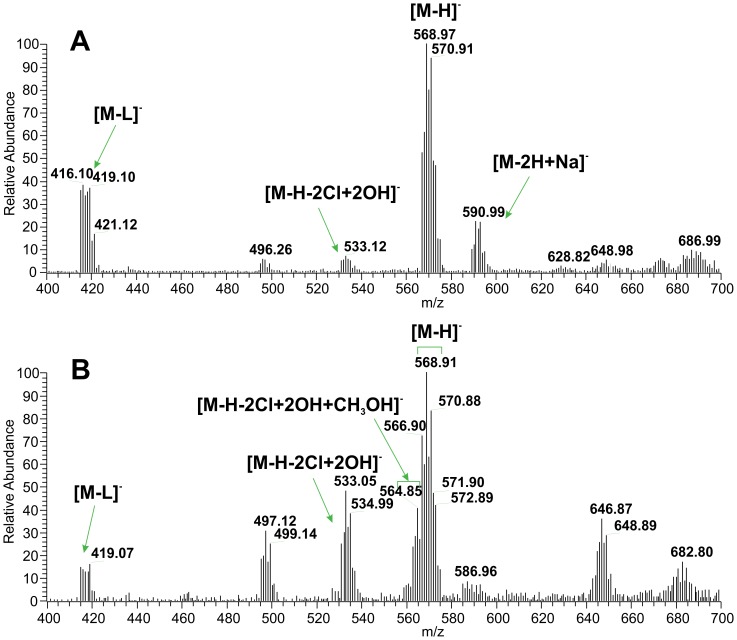
The ESI-MS spectra of the complex 1. The studied complex was dissolved in water/methanol solution (10 µM, 1∶1 v/v) and measured 12 h (A) and 1 month after the preparation (B) using the negative ionization mode.

In addition, the similar ESI mass spectrometry experiment was arranged to test the stability of the complex **1** in water-containing solution, *i.e.* in a water/methanol mixture (10 µM, 1∶1 v/v) in this case. The attention was drawn towards the possible formation of intermediates which could (at least theoretically) facilitate the interaction of the prepared complexes with target biomolecules (*e.g.* parts of the proteasome or DNA), and indeed, a very slow time-dependent accrual in the relative intensity of ions corresponding to hydrolysis products (Figure 3), dominantly the species at *m/z* ^−^ 533.05, together with *m/z* ^−^ 564.85 corresponding to the ionic species [M-H-2Cl+2OH]^−^, and [M-H-2Cl+2OH+CH_3_OH]^−^, respectively, was evident in the mass spectra obtained in the negative ionization mode. In comparison to that, the inverse dependence of intensity on the time was found for another type of intermediate observed at *m/z* ^−^ 416.10, corresponding (according to mass and isotopic distribution) to the ionic species [M-L]^−^; L = *3Cl*aza.

### ESI-MS Interaction Studies of 1 with Cysteine and Reduced Glutathione

In an effort to describe the reactivity of the prepared complexes with the major sulphur-containing biomolecules found in the human plasma, the methanolic solution of the complex **1**, as a representative example, was mixed with the mixture of L-cysteine (Cys) and reduced glutathione (GSH) in water in the physiological concentrations. The macroscopic appearance (a colourless solution without any traces of precipitation) of the solution stayed the same during the whole duration of the experiment. The analysis of mass spectra (and that applies to all measured data) did not reveal the evidence that the prepared complexes could be able to interact with physiological levels of main sulphur-containing biomolecules (Cys and GSH). We did not detect any species whose mass corresponds to the adduct of the fragments or hydrolysis products of the studied platinum(II) complex with Cys and/or GSH even one month after the preparation of the mixture. In fact, only the primal pseudomolecular ion at *m/z* 619.91, corresponding (according to mass and isotopic distribution) to the ionic species [M-H-2Cl+CH_3_OH+5H_2_O]^+^, was clearly present in the mass spectra of the analysed mixture (see Figure S1 in File S1).

### ITC Interaction Studies with Cysteine, Reduced Glutathione and Human Serum Albumin

The ITC experiments for the representative complex **1** and *cisplatin* were performed to demonstrate their ability to interact with Cys, GSH and human serum albumin (HSA) as well as differences between their interaction.

The interaction with biomolecules, such as the above-mentioned reduced glutathione or cysteine, is one of the crucial features of antitumour effective platinum(II) complexes [28]. Since any covalent interactions of the complex **1** with GSH and Cys were not observed by means of ESI-MS, some kind of interactions of the studied complexes with GSH were detected by Uv-Vis spectroscopy in our previous work [24]. Therefore, we decided to use ITC as a very sensitive tool for the investigation and thermodynamic characterization of the interaction (unnecessary covalent from the principle of ITC) with various biomolecules [29], [30]. We performed analogical experiments for the representative complex **1** and *cisplatin* to demonstrate the differences between both platinum(II) dichlorido complexes. An interaction of the complex **1** with both the cysteine and GSH was observed by ITC (see Figure S2 in File S1). Low solubility of the complex **1** in the medium used did not allow us to improve the data quality (in the case of more soluble *cisplatin*, we used the same conditions to make the obtained results on both compounds comparable). The data were fitted by two- (GSH) and three-site (cysteine) sequential binding model to give the following results: *K*_1_ = (1.04±0.10)×10^5^ M^−1^, *ΔH*_1_ = 4.4±0.5 kcal/mol, *ΔS*_1_ = 37.6 cal mol^−1^ K^−1^, *K*_2_ = (9.06±0.60)×10^4^ M^−1^, *ΔH*_2_ = −6.3±0.2 kcal/mol, *ΔS*_2_ = 1.87 cal mol^−1^ K^−1^, *K*_3_ = (9.93±0.63)×10^4^ M^−1^, *ΔH*_3_ = 25.4±2.0 kcal/mol, *ΔS*_3_ = 107 cal mol^−1^ K^−1^ for titration of cysteine and *K*_1_ = (4.32±0.38)×10^4^ M^−1^, *ΔH*_1_ = −0.8±0.1 kcal/mol, *ΔS*_1_ = 18.4 cal mol^−1^ K^−1^, *K*_2_ = (6.99±0.59)×10^4^ M^−1^, *ΔH*_2_ = 4.4±0.2 kcal/mol, *ΔS*_2_ = 36.7 cal mol^−1^ K^−1^ for GSH. In the case of *cisplatin*, the best-fitted results were obtained by three- (GSH) and four-site (cysteine) sequential binding model with the following results: *K*_1_ = (3.71±0.20)×10^5^ M^−1^, *ΔH*_1_ = 7.6±0.3 kcal/mol, *ΔS*_1_ = 50.7 cal mol^−1^ K^−1^, *K*_2_ = (1.18±0.10)×10^5^ M^−1^, *ΔH*_2_ = 2.3±0.2 kcal/mol, *ΔS*_2_ = 30.9 cal mol^−1^ K^−1^, *K*_3_ = (3.97±0.35)×10^4^ M^−1^, *ΔH*_3_ = 44.9±9.4 kcal/mol, *ΔS*_3_ = 169 cal mol^−1^ K^−1^, *K*_4_ = (4.90±0.47)×10^4^ M^−1^, *ΔH*_4_ = −88.8±15.4 kcal/mol, *ΔS*_4_ = −271 cal mol^−1^ K^−1^ for titration of cysteine and *K*_1_ = (3.82±0.34)×10^5^ M^−1^, *ΔH*_1_ = −3.2±0.1 kcal/mol, *ΔS*_1_ = 14.9 cal mol^−1^ K^−1^, *K*_2_ = (3.62±0.36)×10^4^ M^−1^, *ΔH*_2_ = 2.7±0.5 kcal/mol, *ΔS*_2_ = 29.6 cal mol^−1^ K^−1^, *K*_3_ = (6.72±0.67)×10^4^ M^−1^, *ΔH*_3_ = −9.6±0.7 kcal/mol, *ΔS*_3_ = −9.5 cal mol^−1^ K^−1^ for GSH. A comparison of the data obtained on **1** and *cisplatin* indicated different non-covalent interactions of these platinum(II) dichlorido complexes with cysteine and GSH (see Figure S2 in File S1), but due to the above-mentioned reasons regarding solubility we were unable to compare the systems from thermodynamic point of view.

Serum proteins, including human serum albumin (HSA), are well-known to perform transport intracellular and delivery of the platinum(II) metallodrugs to the tumour tissues [31], [32]. It has been also proved that an interaction of some platinum(II) complexes with albumin could result in enhancement of antitumour activity [33]. Therefore the study of a complex (**1** and *cisplatin* in the case of this work) interaction with HSA should be carried out for complete description of biological properties of the studied substance. Again, we used ITC as a sensitive and capable thermodynamic characterization method suitable for the description of a platinum complex interaction with serum albumin (see Figure S2 in File S1). The data for the complex **1** were fitted to a two-site model resulting in the following thermodynamic parameters: *n*_1_ = 33.4±1.70, *K*_1_ = (1.37±0.18)×10^4^ M^−1^, *ΔH*_1_ = −20.0±2.3 kcal/mol, *ΔS*_1_ = −47.2 cal mol^−1^ K^−1^, *n*_2_ = 28.4±1.87, *K*_2_ =  (1.67±0.20)×10^7^ M^−1^, *ΔH*_2_ = −10.8±0.0 kcal/mol, *ΔS*_2_ = −2.49 cal mol^−1^ K^−1^ (for *cisplatin* the data *K*_1_ =  (−1.06±0.52)×10^3^ M^−1^, *ΔH*_1_ = −29.5±1.4 kcal/mol, *K*_2_ =  (7.89±3.20)×10^4^ M^−1^, *ΔH*_2_ = 31.8±1.3 kcal/mol, *ΔS*_2_ = 1.07 kcal mol^−1^ K^−1^, *K*_3_ =  (−4.46±0.21)×10^3^ M^−1^, *ΔH*_3_ = −27.8±0.7 kcal/mol were obtained by a three-site sequential binding model). In other words, the ITC results indicated two different binding sites of the complex **1** on albumin and different type of non-covalent interaction of the complex **1** in comparison with *cisplatin*. Similar results, but with only one-fold difference between the binding constants *K*_1_ and *K*_2_, were reported for (to the best of our knowledge) the only platinum complex, whose interaction with HSA was studied by ITC [34]. The presumption that the difference in *K*_n_ could be caused by the conformation changes of HSA after binding of the complex to the first binding site, has to be taken into account as well.

### *In Vitro* Antitumour Activity

**Figure 4:**
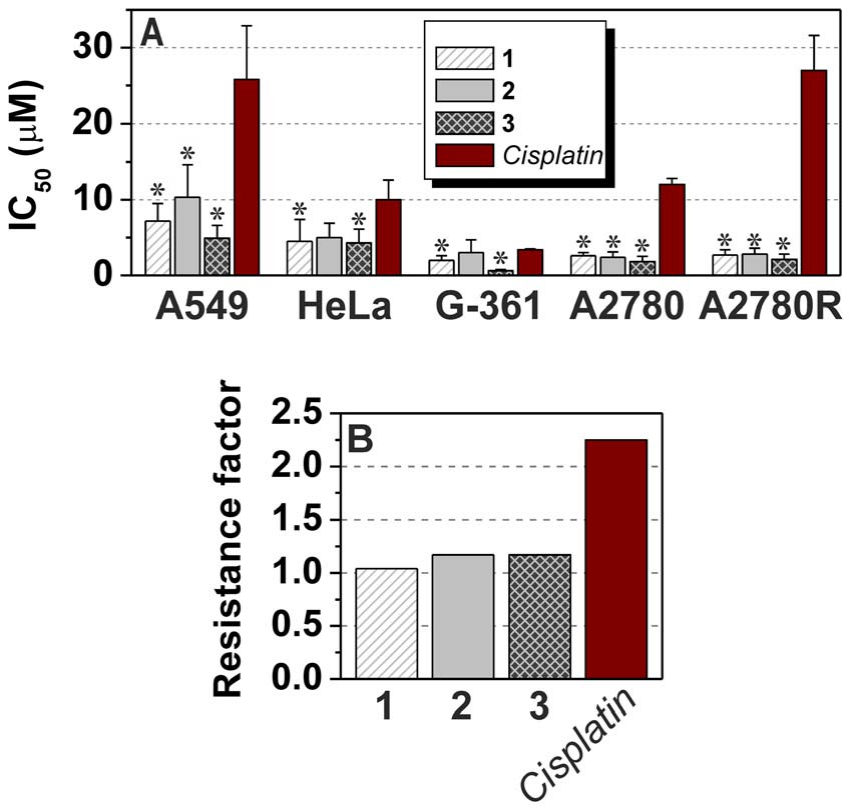
The *in vitro* antitumour activity testing results. The data were obtained by the MTT assay against human lung carcinoma A549, cervix epithelial carcinoma HeLa, malignant melanoma G-361, ovarian carcinoma A2780 and *cisplatin*-resistant ovarian carcinoma A2780R cell lines for **1**–**3** and *cisplatin*. The cells were exposed to the compounds for 24 h. Measurements were performed in triplicate and each cytotoxicity experiment was repeated three times. The given IC_50_±S.D. (µM) values represent an arithmetic mean. The asterisk (*) denotes a significant difference (*p*<0.05) between the studied complexes and *cisplatin* (A); the plot of resistance factors calculated as IC_50_(A2780R)/IC_50_(A2780) for **1**–**3** and *cisplatin* (B).

The complexes **1**–**3** were studied for their antitumour activity *in vitro* against lung carcinoma A549, cervix epithelial carcinoma HeLa, malignant melanoma G-361, ovarian carcinoma A2780 and *cisplatin*-resistant ovarian carcinoma A2780R human cancer cell lines, commonly used in the antitumour activity testing of platinum(II) complexes [35]–[38]. The results are given in Figure 4 and Table 1.

**Table 1:** *In vitro* antitumour activity given as IC_50_ ± S.D. in µM of the complexes **1**–**3** and *cisplatin*.

Cell line	1	2	3	*Cisplatin*	Reference
A549	7.2±2.3 *	10.3±4.3 *	4.9±1.7 *	25.8±7.1	This work
HeLa	4.5±2.9 *	5.0±1.9	4.3±1.8 *	10.0±2.6	This work
G-361	2.0±0.6 *	3.0±1.7	0.6±0.2 *	3.4±0.1	This work
A2780	2.6±0.4 *	2.4±0.7 *	1.8±0.7 *	12.0±0.8	This work
A2780R	2.7±0.7 *	2.8±0.8 *	2.1±0.8 *	27.0±4.6	This work
MCF7	3.4±0.3 *	8.0±0.9	2.0±0.2 *	19.6±4.3	[23]
HOS	3.8±0.1 *	3.9±0.2 *	2.5±0.1 *	34.2±6.4	[23]
LNCaP	3.3±0.7	3.8±1.3	1.5±0.4	3.8±1.5	[23]

Asterisks (*) symbolize significant difference (*p*<0.05) in *in vitro* antitumour activity of **1**–**3** as compared to *cisplatin*.

**Figure 5:**
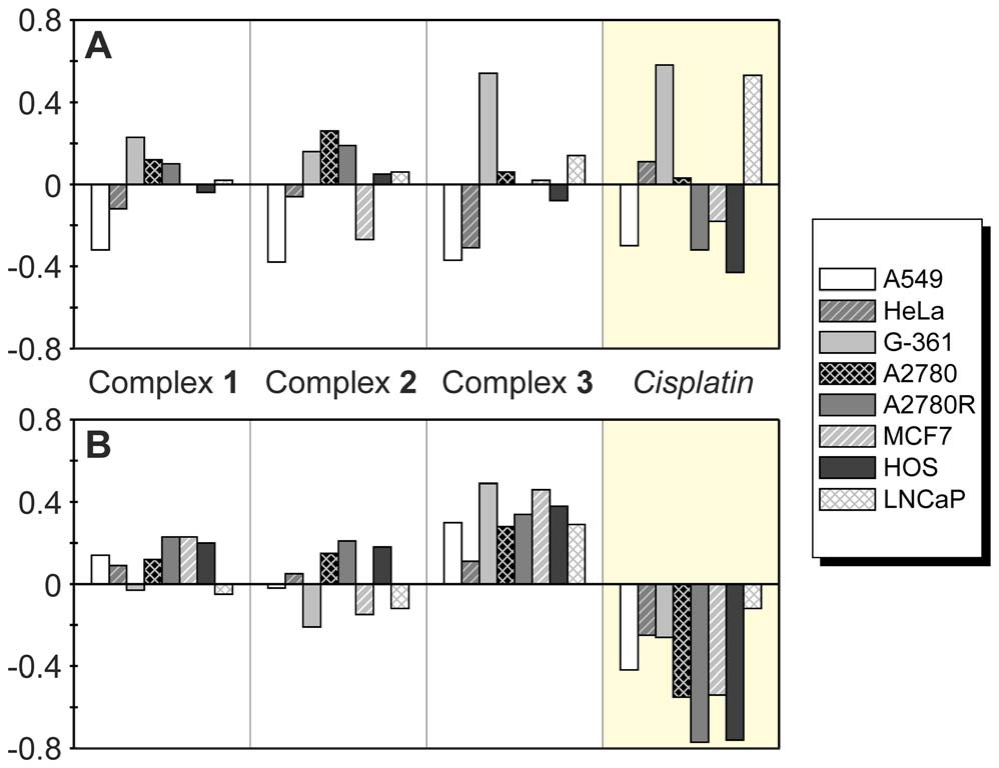
The results of *in vitro* antitumour activity on logarithmic scale. The plot representing the differences from the mean logIC_50_ obtained for the individual compounds (**1**, **2**, **3** and *cisplatin*) on eight human cancer cell lines reported in this work (A549, HeLa, G-361, A2780 and A2780R) and in [23] (MCF7, HOS and LNCaP), where the positive values show the sensitivity of the particular cell line, while the negative ones relate to the resistance of the cancer cell line to the action of the platinum(II) complex (A) and the differences from the mean logIC_50_ obtained on the individual human cancer cell lines reported in this work (A549, HeLa, G-361, A2780 and A2780R) and in [23] (MCF7, HOS and LNCaP) (B).

The complexes **1**–**3** exceeded the antitumour activity *in vitro* of *cisplatin* against all the employed cancer cell lines as they were found to be 3.6-, 2.5- and 5.3-times (A549), 2.2-, 2.0- and 2.3-times (HeLa), 1.7-, 1.1- and 5.7-times (G-361), 4.6-, 5.0- and 6.7-times (A2780) and 10.0-, 9.6- and 12.9-times (A2780R) more effective than *cisplatin*. The complex **3** was the most active substance determined by the *in vitro* experiments with IC_50_ values lower than those of *cisplatin* as well as both the complexes **1** and **2**. The complexes **1** and **3** were significantly more antitumour active *in vitro* (ANOVA, *p*<0.05) against all the cell lines as compared with *cisplatin*, while the same conclusion can be made for the complex **2** only against the A549, A2780 and A2780R cell lines (Figure 4, Table 1). These results indirectly proved that the studied platinum(II) complexes with 7-azaindoles are able to overcome intrinsic resistance to *cisplatin* on the A549, A2780 and A2780R (**1**–**3**), and HeLa and G-361 (**1**, **3**) human cancer cell lines *in vitro*. To support this statement, we used a comparison of logIC_50_ (see Table S1 in File S1) of the complexes **1**–**3** and *cisplatin*, particularly the differences between the mean logIC_50_ observed for individual substances on all cancer cell lines reported in this work and in [23], and logIC_50_ of the individual substances against the concrete cell line (Figure 5A), and the differences between the mean logIC_50_ obtained on individual human cancer cell lines and logIC_50_ of the individual complex (Figure 5B), to demonstrate the sensitivity or resistance of the cancer cell to the action of the studied complexes in comparison with the others including *cisplatin* [39], [40].

The antitumour activity *in vitro* of the complexes **1**–**3** can be evaluated also by means of the resistance factors, since the substances were tested on both *cisplatin*-sensitive (A2780) and resistant (A2780R) ovarian carcinoma cell lines (Figure 4). The resistance factors, calculated as IC_50_(A2780R)/IC_50_(A2780), equal 1.04 (**1**), 1.17 (**2**), 1.17 (**3**) and 2.25 (*cisplatin*), which show on the ability of the complexes **1**–**3** to avoid also the acquired type of cancer cell resistance against *cisplatin*. This feature of the studied complexes is in good agreement with the above-discussed ability of the studied complexes to overcome an interaction with the sulphur-containing biomolecules, which is well-known as one of the crucial factors decreasing the resistance of various tumours to the action of platinum-based drugs [11]–[13].

To conclude the *in vitro* antitumour activity testing of the complexes **1**–**3** on the human cancer cell lines, it has to be stated that they were recently tested for their antitumour activity *in vitro* against three others human cancer cell lines (*i.e.* eight human cancer cell lines in total), namely breast adenocarcinoma MCF7 (IC_50_ = 3.4, 8.0, and 2.0 µM for **1**–**3**, respectively), osteosarcoma HOS (IC_50_ = 3.8, 3.9, and 2.5 µM for **1**–**3**, respectively) and prostate adenocarcinoma LNCaP (IC_50_ = 3.3, 3.8, and 1.5 µM for **1**–**3**, respectively) [23]. Two of the studied complexes (**1**, **3**) have significantly higher *in vitro* antitumour activity (*p*<0.05) than *cisplatin* against all eight human cancer cell lines, while the complex **2** only on the A549, A2780, A2780R and HOS cells. Although the testing of the platinum complexes against five or more human cancer cell lines is quite common in the literature [40]–[42], the markedly higher antitumour effect than control (*cisplatin* in this work as well as in most of similarly focused paper) obtained on all the cell lines may be considered as unique to the best of our knowledge.

### *In vivo* Antitumour Activity

**Figure 6:**
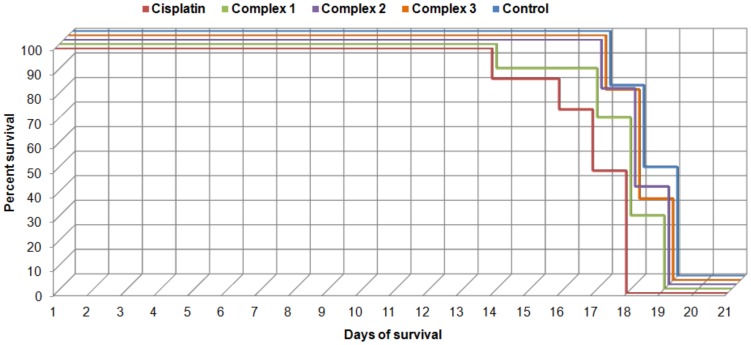
The Kaplan-Meier curves depicting the percentage of survival of animals in the individual groups.

The *in vivo* antitumour activity of the complexes **1–3** and the reference drug *cisplatin* was tested on the mouse model of lymphocytic leukemia (L1210) using the complexes as therapeutic agents in the 7-day dosing schedule (3 mg/kg, *i.p.*) following the previous 10-day initiation period in which the primary tumours formed (examined by palpation). The percentages of mean survival time, %T/C defined as the ratio of the mean survival time of the treated animal groups (T) divided by the mean survival time of the untreated control group (C), were calculated and are shown in Table 2 and Figure 6. It should be noted that despite the relatively large differences of the absolute %T/C values between the *cisplatin* group and the others experimental groups (**1**–**3**), none of these results proved to be significantly different (*p*<0.01). This is not a surprising result, because to reach the similar results of %T/C between two antitumour active compounds, they usually have to share also a portion of similarity in toxicological, pharmacodynamic, and pharmacokinetic parameters. It seems likely, that this was not a case in the described experiment. Even if the tested complexes showed no significant extension of the mean survival time in comparison to the control group, the decision about the overall antitumour activity of tested complexes is much more complex process. That is why we chose a multiparametric semiquantitative approach to evaluate the overall antitumour activity of both the prepared complexes and *cisplatin*, used as a standard.

**Table 2:** Selected parameters obtained from the pharmacological part of the *in vivo* L1210 antitumour activity model.

Compound	Control	1	2	3	*Cisplatin*
Body weight ± S.E. (g)	24.76±1.28	25.06±0.85	23.08±0.76	22.60±1.49	14.80±0.43
Weight of the cancer tissues ± S.E. (g)	5.06±0.58	5.09±0.18	4.75±0.15	4.23±0.40	1.50±0.01
% of tumour tissues ± S.E.	20.45±2.35	20.31±0.72	20.59±0.63	18.74±1.78	10.13±0.05
Mean survival time ±S.E. (days)	18.2±0.3	17.7±0.5	18.2±0.2	18.1±0.3	17.0±0.5
% T/C	100.0	97.1	100.0	99.4	93.3

Other parameters, which were determined *post mortem*, comprise the weight of the tumour tissue and its weight ratio to the whole body weight (see Table 2). Both these parameters proved the aggressive cytotoxic action of *cisplatin* (elimination of more than 50% of tumour tissues, as compared to control group), while the effect of the complexes **1**–**3** was much more gradual (the complexes **2** and **3** caused the 2%, and 8% decrease of tumour tissue weight, respectively).

The overall health status observations made during the whole time of the experiment showed that the animals in the groups treated by the complexes **1–3** showed no signs of toxicity or changes of normal behavior up to the 18^th^ day of the experiment (in average the last day of the experiment), while the animals treated with *cisplatin* showed all known side effects of this therapy, *i.e*. loss of weight, fatigue, loss of appetite, various types of aberrant behavior. With respect to the given results we can conclude that the lower toxicity of the complexes **1–3** and less aggressive effect against tumour cells have to be considered as more beneficial for the treated animals than far more aggressive action of *cisplatin*.

In addition to the macroscopic observations, much more information was obtained from the methods that evaluated the mechanisms of cytotoxicity, starting from the histological and histochemical observations, followed by the ELISA and Westernblot determination of the expression of selected proteins.

### Macroscopic and Histological Observations

All the animals implanted with the cell line L1210 expressed significant tumour infiltration of the abdominal cavity, peritoneal infiltration and well circumscribed tumours in the place of application, which were identified as anaplastic lymphoma. In all the animals, these tumours proliferated in the visceral fatty tissue and in the gonadal area, and formed well circumscribed focuses. Tumour cell dissemination of the diffuse type was found in GALT (gut-associated lymphoid tissue), the proliferation of the tumour tissues was found in the ileocaecal area and mesocolon. This type of tumour was identified according the WHO classification as diffuse B-lymphoma [43]. The infiltration by tumour cells was observed in several different organ preparations, but no metastatic focuses were identified. This observation is in agreement with the unchanged level of MMPs (see Figure S3 in File S1), which are responsible, at least in part, for metastasis formation [44].

### Evaluation of Cellular Processes and Semiquantitative Immunohistochemical Detection of Selected Proteins in Tumour Tissue Samples

The results of antiproliferative or pro-apoptotic activity, and necrosis-induction and other physiopathological processes, as determined by the histological and histochemical methods, induced by the tested compounds are summarized in Table 3. As a parameter that indicated the overall state of tumour regression, the reactivity index was calculated as the ratio of atypical mitoses to the apoptoses in one view field of a microscope at 200× magnification. The reactivity index demonstrates quite well the reactivity of the tumour tissue to the chemotherapy and its possible outcomes, *i.e.* the probability of further regression of the tumour tissues or, on the other hand, the possibility to restore the earlier rates of its proliferation leading to the impending death.

**Table 3:** Selected parameters obtained from the histological and immunohistochemical analyses of the *in vivo* antiproliferative assay.

Compound	Control	1	2	3	*Cisplatin*
Necrosis	2	2	2	3	4
Hemorrhage	1	3	3	4	3
Mitotic Index	56	48	44	36	23
Caspase 8	3	2	2	4	3
Caspase 3	1	4	3	4	1
p53	2	2	2	4	4
TNF-α	2	0	0	1	0
Total Score	11	13	12	20	15
Reactivity Index	5.60	3.69	3.67	1.71	1.35

The semi-quantitative evaluation of the tumour reactivity revealed, as expected from the lowering of the tumour tissue weight, the highest antiproliferative activity in the *cisplatin*-treated group of animals with the reactivity index of 1.35. The relatively low mitotic activity was accompanied by massive necroses and hemorrhage, which might be responsible for the induction of the inflammatory response documented by the higher detection of the pro-inflammatory cytokine TNF-α. The induction of the pro-inflammatory cellular responses was recently associated also with its nephrotoxicity [45]. The reactivity index calculated for the complex **3**-treated group, which did not correlate proportionally with the decrease of tumour tissue mass, was found to be very positive and perspective. Nevertheless, its absolute value of 1.71 indicates that the complex **3** stimulates all the cellular signs of tumour regression, even if its onset of action is in comparison with *cisplatin* significantly slower. In the complex **1**- and **2**-treated groups, there were also found signs of tumour regression (reactivity index was 3.69, and 3.67, respectively), in this case, however, their impact on the reduction of the tumour tissue mass was insignificant. Other parameters semi-quantitatively determined in the tissue samples, are in agreement with the above mentioned overall evaluation on the basis of the reactivity index and their incidence correlate quite well with its values. These preliminary results could serve as the basis for further *in vivo* studies of the most promising compound (*i.e.* the complex **3**) on the solid tumour models.

### ELISA and Westernblot Determination of the Expression of Selected Proteins

**Figure 7:**
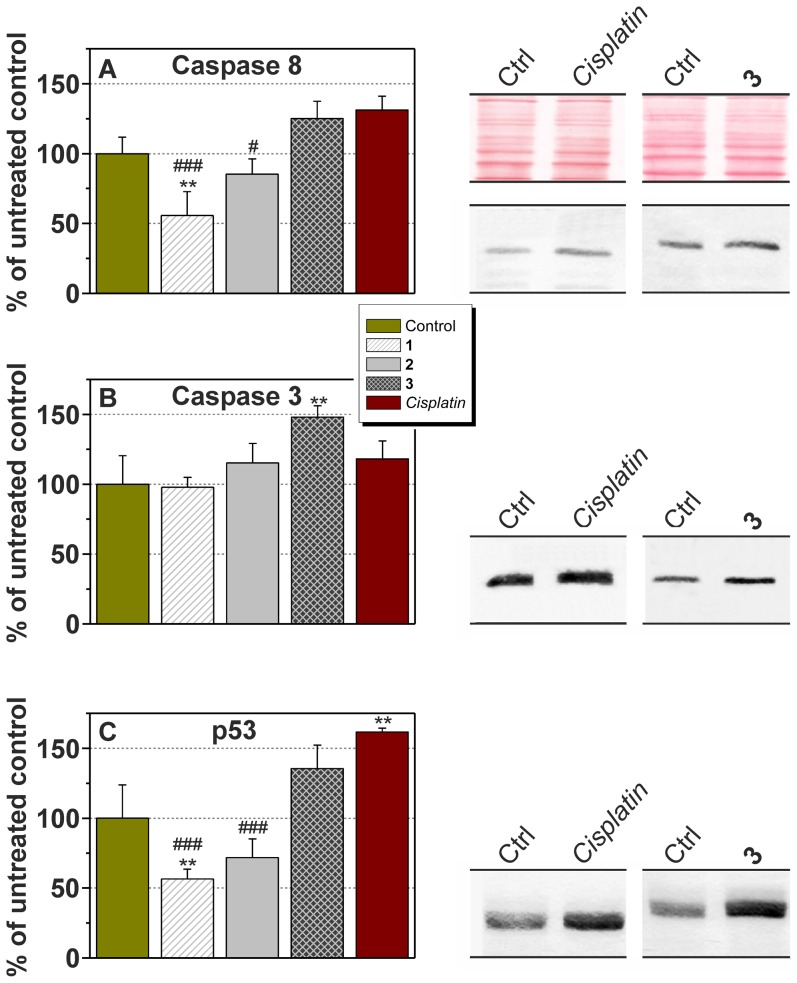
The results of the studies of Casp-8, Casp-3 and p53 expression. (A): The amount of the active form of Casp-8 (p18) was detected by Westernblot and immunodetection. The results are presented as mean ± S.E. ** significant difference in comparison to untreated cells (*p<*0.01), # significant difference in comparison to *cisplatin*-treated cells (*p<*0.05), ### significant difference in comparison to *cisplatin*-treated cells (*p<*0.001). (B): the amount of the active form of Casp-3 (p17) was detected by Westernblot and immunodetection. The results are presented as mean ± S.E. ** significant difference in comparison to untreated cells (*p<*0.01). (C): Amount of p53 was detected by Westernblot and immunodetection. The results are presented as mean ± S.E. ** significant difference in comparison to untreated cells (*p<*0.01), ### significant difference in comparison to *cisplat*in-treated cells (*p<*0.001).

A tumour blood supplementation is very important for its growth. Hence, the vascular endothelial growth factor A (VEGF-A) was quantified in tumour lysates. No significant change was observed for this cytokine, only the complex **1** slightly decreased its amount in comparison with the untreated samples (see Figure S4 in File S1). A level of the initiator caspase 8 (Casp-8), effector caspase 3 (Casp-3), and regulatory protein p53 was also measured. The complex **1** significantly decreased the amount of the active form of Casp-8 (p18) by the factor of 1.8 in comparison with the untreated control tumours (Figure 7A). The complex **2** also reduced p18, but only 1.2-times without any statistical significance. On the other hand, the complex **3** and *cisplatin* slightly increased the level of active Casp-8 by the factor of 1.3. In comparison to *cisplatin*, the complexes **1** and **2** showed significant diminution of the Casp-8 level by the factor of 2.4, and 1.5, respectively. The complex **3** was the only compound which was able to significantly increase the level of the active form of the effector Casp-3 (Figure 7B), concretely 1.5-times in comparison to the untreated tumours. The complex **2** and *cisplatin* only weakly raised its amount by the factor of 1.2 and the complex **1** did not have any effect. The expression pattern for p53 was similar to Casp-8. The complex **1** significantly decreased the level of p53 by the factor of 1.8 in comparison to the untreated tumours (Figure 7C). The moderate diminution of p53 level was also detected for the complex **2** (1.4-times). An increase of the p53 level was observed in the case of the complex **3** (1.4-times) and *cisplatin* (1.6-times). Finally, all the groups treated by the platinum(II) complexes showed lower mitotic index (MI; lower MI indicates tumour-static or tumour-toxic activity of the given complexes), which indicates tumour regression, in comparison with untreated control, in the order of its absolute value: *cisplatin* <**3**<**2**<**1**. In other words, the obtained results showed on different action of the complexes **1** and **2** as compared with the complex **3** and *cisplatin* in accordance with the above-mentioned results of both *in vitro* and *in vivo* studies.

It is a well-established fact that antitumour active platinum(II) complexes covalently bind to the DNA molecule [46]. This mode of action was, together with notably higher cell-uptake and DNA platination as compared with *cisplatin*, also proved within the mechanistic studies performed very recently on the herein reported complexes with 7-azaindoles [23], [24]. Platination of DNA molecule induces DNA damage which results in a cell cycle arrest or in apoptosis [47]. This effect is usually caused by the stabilization and activation of the transcription factor p53, which recognizes damaged DNA and is able to trigger the intrinsic (mitochondrial) apoptotic pathway in the case of massive DNA damage [48]. This mechanism is in concordance with our results, since we detected non-significantly higher amounts of p53 in tumours treated by both the most *in vivo* active compounds - *cisplatin* and the complex **3**. On the other hand, the complex **1** significantly decreased the p53 expression, but the amount of active effector Casp-3 remained unchanged. This effect could be caused by the activation of Casp-3 by p53-independent pathways, *e.g.* via p73 activation [49]. The complexes **1** and **2** also attenuated activation of the initiator Casp-8, which might indicate the triggering of apoptosis *via* an intrinsic pathway rather than extrinsic pathway [50]. Higher amounts of active Casp-3 after the complex **3** and *cisplatin* treatment correspond with TNF-α positivity observed in histological preparations. It has been described that macrophages surrounding cancer cells are able to produce antitumour cytokines, *e.g.* TNF-α and FasL, after *cisplatin* treatment *in vitro* [51]. These cytokines subsequently trigger apoptosis via activation of TNF-α and FasL receptors. The higher production of TNF-α, and the related inflammatory reaction related with it, is probably responsible for significantly elevated necrosis and hemorrhage after *cisplatin* and the complex **3** treatments.

## Conclusions

In conclusion, this work represents a substantial contribution to the broad and systematic study of *cisplatin*-derived complexes involving the 7-azaindole derivatives (*n*aza) for the identification of notable biological activities and molecular mechanisms interconnected with these activities. The presented data elaborate previous promising results of *in vitro* cytotoxicity screening carried out on a series of *cis*-[PtCl_2_(*n*aza)_2_] complexes (**1**–**3**) and underlying mechanisms based on the interaction of the complexes with DNA. In this paper, we extended the scope of *in vitro* cytotoxicity testing to the expanded panel of human cancer cell lines (A549, HeLa, A2780 and *cisplatin*-resistant A2780R, and G-361) and found significant antitumour activity *in vitro* (the IC_50_ values were as low as 1 µM level), which surpassed that of *cisplatin* considerably. The complexes **1**–**3** also proved to be able to avoid the acquired resistance of the A2780R cell line to *cisplatin*. The *in vitro* studies, which strengthened the evidence in the perspective of advanced testing of biological activities, were followed by *in vivo* and *ex vivo* trials based on the mouse L1210 lymphocytic leukemia model. The pharmacological observations have been complemented by the histological and immunohistochemical evaluations of isolated cancerous tissues and the expression of selected key proteins, associated with the tumour growth progression and induction of apoptosis (*i.e.* caspases 3 and 8, p53, VEGF-A were determined by ELISA and Western blot methods). The complexes **1**–**3** (applied at the same dose as *cisplatin*) showed that their effect on the reduction of cancerous tissues volume is markedly lower than that of *cisplatin*, however, they also proved to cause less serious adverse effects on the healthy tissues and the health status of the treated mice. The results of *ex vivo* assays revealed that above all others, the complex **3** was also able to effectively modulate the levels of active forms of caspases 3 and 8, and the transcription factor p53, and thus activate the intrinsic (mitochondrial) pathway of apoptosis. These results, unequivocally confirming the highest antitumour effect of the complex **3**, were supported both by the histological and immunohistochemical observations. The overall positive results of antitumour activity testing were supported by the results of stability and interaction studies, which indicated the ability of the tested complexes to resist the formation of covalent adducts with low molecular sulfur-containing biomolecules in the biologically applicable media. The presented results unambiguously demonstrate that there is still a room for improvement of pharmacological properties of antitumour *cis*-dichloridoplatinum(II) complexes using the synthetic approach, based on the substitution of carrier ligands and that the 7-azaindole might serve as a viable basis for such studies.

## Supporting Information

File S1 **Supporting information.** The ^1^H, ^13^C, ^15^N and ^195^Pt NMR data assigned to *trans*-[PtCl_2_(*n*aza)_2_] complexes (**1t**, **2t**, **3t**) detected as an impurity of the studied *cis*-[PtCl_2_(*n*aza)_2_] complexes, the ^1^H, ^13^C, ^15^N and ^195^Pt NMR data for the products of hydrolysis of **1** and **3** in DMF-*d_7_*/H_2_O mixture (**1 h** and **3 h**). **Figure S1**. The ESI-mass spectrum of the mixture of the complex **1** with cysteine and glutathione measured one month after the mixing of the components. **Figure S2**. ITC results showing the heat released during the titration of cysteine, GSH and HSA by 1 or *cisplatin* and the binding isotherms. **Figure S3**. The effect of **1**–**3** and *cisplatin* on MMPs secretion in isolated tumours. **Figure S4**. The effect of **1**–**3** and *cisplatin* on VEGF-A secretion in isolated tumours. **Table S1**. The values of logIC_50_ (µM) for the complexes **1**–**3** and *cisplatin*.
(DOCX)
