# Supplementary material for: Pharmacological and Molecular Effects of Platinum(II) Complexes Involving 7-Azaindole Derivatives
Source: PLoS One. 2014 Mar 6;9(3):e90341. doi: 10.1371/journal.pone.0090341 (PMC3948342; doi:10.1371/journal.pone.0090341)
Supplement: File S1 — Supporting information. The 1H, 13C, 15N and 195Pt NMR data assigned to trans-[PtCl2(naza)2] complexes (1t, 2t, 3t) detected as an impurity of the studied cis-[PtCl2(naza)2] complexes, the 1H, 13C, 15N and 195Pt NMR data for the products of hydrolysis of 1 and 3 in DMF-d7/H2O mixture (1 h and 3 h). Figure S1. The ESI-mass spectrum of the mixture of the complex 1 with cysteine and glutathione measured one month after the mixing of the components. Figure S2. ITC results showing the heat released during the titration of cysteine, GSH and HSA by 1 or cisplatin and the binding isotherms. Figure S3. The effect of 1–3 and cisplatin on MMPs secretion in isolated tumours. Figure S4. The effect of 1–3 and cisplatin on VEGF-A secretion in isolated tumours. Table S1. The values of logIC50 (µM) for the complexes 1–3 and cisplatin. (DOCX) [file pone.0090341.s001.docx]

# Pharmacological and molecular effects of platinum(II) complexes involving 7-azaindole derivatives

Pavel Štarha^1^, Jan Hošek^1^, Ján Vančo^1^, Zdeněk Dvořák^2^, Pavel Suchý Jr.^3^, Igor Popa^1^, Gabriela Pražanová^3^, Zdeněk Trávníček^1^*

**^1^** Department of Inorganic Chemistry, Regional Centre of Advanced Technologies and Materials, Faculty of Science, Palacký University, Olomouc, Czech Republic, **^2^**Department of Cell Biology and Genetics, Regional Centre of Advanced Technologies and Materials, Faculty of Science, Palacký University, Olomouc, Czech Republic,

**^3^**Department of Human Pharmacology and Toxicology, Faculty of Pharmacy, University of Veterinary and Pharmaceutical Sciences Brno, Brno, Czech Republic

Email addresses: PŠ (pavel.starha@upol.cz), JH (jan.hosek@upol.cz), JV (jan.vanco@upol.cz), ZD (zdenek.dvorak@upol.cz), PS (suchypa@vfu.cz), IP (igor.popa@upol.cz), GP (gaba.p@seznam.cz), ZT (zdenek.travnicek@upol.cz).

* Corresponding author: Department of Inorganic Chemistry, Regional Centre of Advanced Technologies and Materials, Faculty of Science, Palacký University, 17. listopadu 12, CZ-771 46 Olomouc, Czech Republic. Phone: +420-585-634-352. Fax: +420-585-634-954. E-mail: zdenek.travnicek@upol.cz.

**NMR spectroscopy**

*Note:* The results of ^1^H, ^13^C, ^15^N and ^195^Pt NMR spectroscopy for the studied *cis*-[PtCl_2_(*n*aza)_2_] complexes (**1**–**3**) are given in Ref. 22. Herein we provide the NMR data assigned to *trans*-[PtCl_2_(*n*aza)_2_] complexes (symbolized **1t**, **2t** and **3t**), which were detected as an impurity of **1**–**3**. The NMR data for the products of hydrolysis of **1** and **3** in DMF-*d_7_*/H_2_O mixture (symbolized **1h** and **3h**) can be also found below.

**1t**: ^1^H NMR (400 MHz, DMF-*d_7_*): δ 13.03 (br, 1H, N1–H), 8.78 (d, 5.6, 1H, C6–H), 8.19 (d, 9.3, 1H, C4–H), 8.06 (s, 1H, C2–H), 7.40 (m, 1H, C5–H). ^13^C NMR (100 MHz, DMF-*d_7_*): δ 147.4 (C6), 145.6 (C7`), 129.0 (C4), 125.0 (C2), 120.7 (C3`), 117.1 (C5), 103.9 (C3). ^15^N NMR (40 MHz, DMF-*d_7_*): δ 142.1 (N1), 168.2 (N7). ^195^Pt NMR (86 MHz, DMF-*d_7_*): δ –2111.7.

**2t**: ^1^H NMR (400 MHz, DMF-*d_7_*): δ 12.93 (br, 1H, N1–H), 8.75 (dd, 5.7, 1.2, 1H, C6–H), 8.06 (d, 2.7, 1H, C2–H), 7.96 (d, 8.5, 1H, C4–H), 7.40 (m, 1H, C5–H). ^13^C NMR (100 MHz, DMF-*d_7_*): δ 147.1 (C6), 147.0 (C7`), 132.3 (C2), 131.5 (C4), 125.4 (C3`), 117.3 (C5), 56.8 (C3). ^15^N NMR (40 MHz, DMF-*d_7_*): δ 150.3 (N1), 168.1 (N7). ^195^Pt NMR (86 MHz, DMF-*d_7_*): δ –2109.6.

**3t**: ^1^H NMR (400MHz, DMF-*d_7_*): δ 13.08 (br, 1H, N1–H), 8.80 (d, 1.8, 1H, C6–H), 8.45 (d, 1.7, 1H, C4–H), 7.88 (t, 3.2, 1H, C2–H), 6.73 (m, 1H, C3–H). ^13^C NMR (100 MHz, DMF-*d_7_*): δ 146.4 (C7`), 145.2 (C6), 133.4 (C4), 130.3 (C2), 124.6 (C3`), 109.4 (C5), 101.6 (C3). ^15^N NMR (40 MHz, DMF-*d_7_*): δ 144.6 (N1), 170.9 (N7). ^195^Pt NMR (86 MHz, DMF-*d_7_*): δ –2099.3.

**1h**: ^1^H NMR (400MHz, DMF-*d_7_*/H_2_O): δ 13.28 (br, 1H, N1–H), 9.00 (dd, 5.8, 0.9, 1H, C6–H), 8.14 (dd, 8.0, 1.3, 1H, C4–H), 7.97 (s, 1H, C2–H), 7.38 (m, 1H, C5–H). ^13^C NMR (100 MHz, DMF-*d_7_*/H_2_O): δ 145.4 (C6), 144.4 (C7`), 128.3 (C4), 123.7 (C2), 119.7 (C3`), 116.8 (C5), 103.2 (C3). ^15^N NMR (40 MHz, DMF-*d_7_*/H_2_O): δ 133.8 (N1), 164.8 (N7). ^195^Pt NMR (86 MHz, DMF-*d_7_*/H_2_O): δ –2133.5.

**3h**: ^1^H NMR (400MHz, DMF-*d_7_*/H_2_O): δ 13.38 (br, 1H, N1-H), 9.24 (d, 1.8, 1H, C6–H), 8.37 (d, 1.9, 1H, C4–H), 7.91 (d, 3.5, 1H, C2–H), 6.74 (d, 3.5, 1H, C3–H). ^13^C NMR (100 MHz, DMF-*d_7_*/H_2_O): δ 145.9 (C7`), 144.2 (C6), 133.1 (C4), 129.0 (C2), 124.0 (C3`), 109.4 (C5), 101.4 (C3). ^15^N NMR (40 MHz, DMF-*d_7_*/H_2_O): δ 139.6 (N1), 170.3 (N7). ^195^Pt NMR (86 MHz, DMF-*d_7_*/H_2_O): δ –2114.0.

**
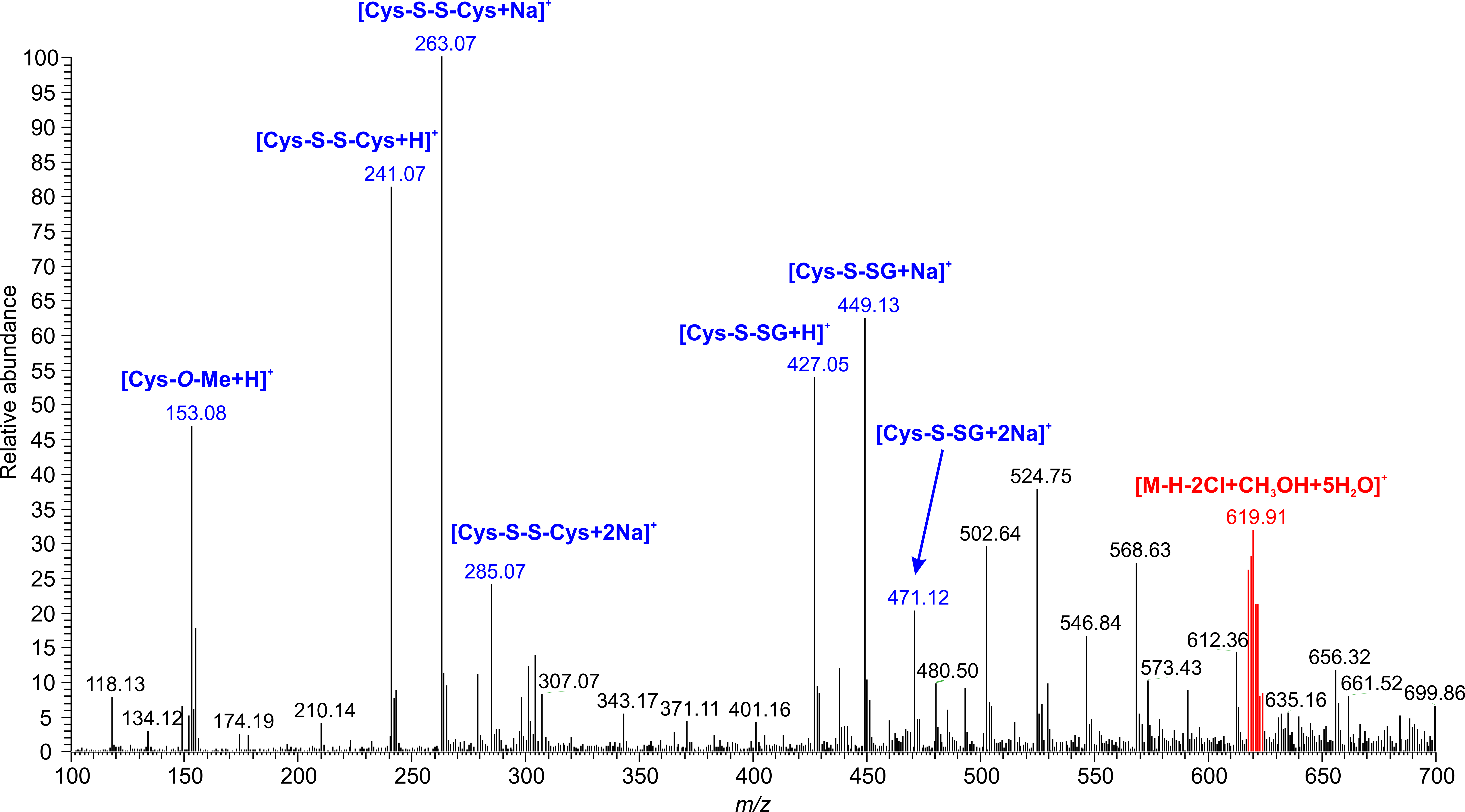
**

**Figure S1.** The ESI-mass spectrum of the mixture of complex 1 with cysteine and glutathione measured one month after the mixing of the components.
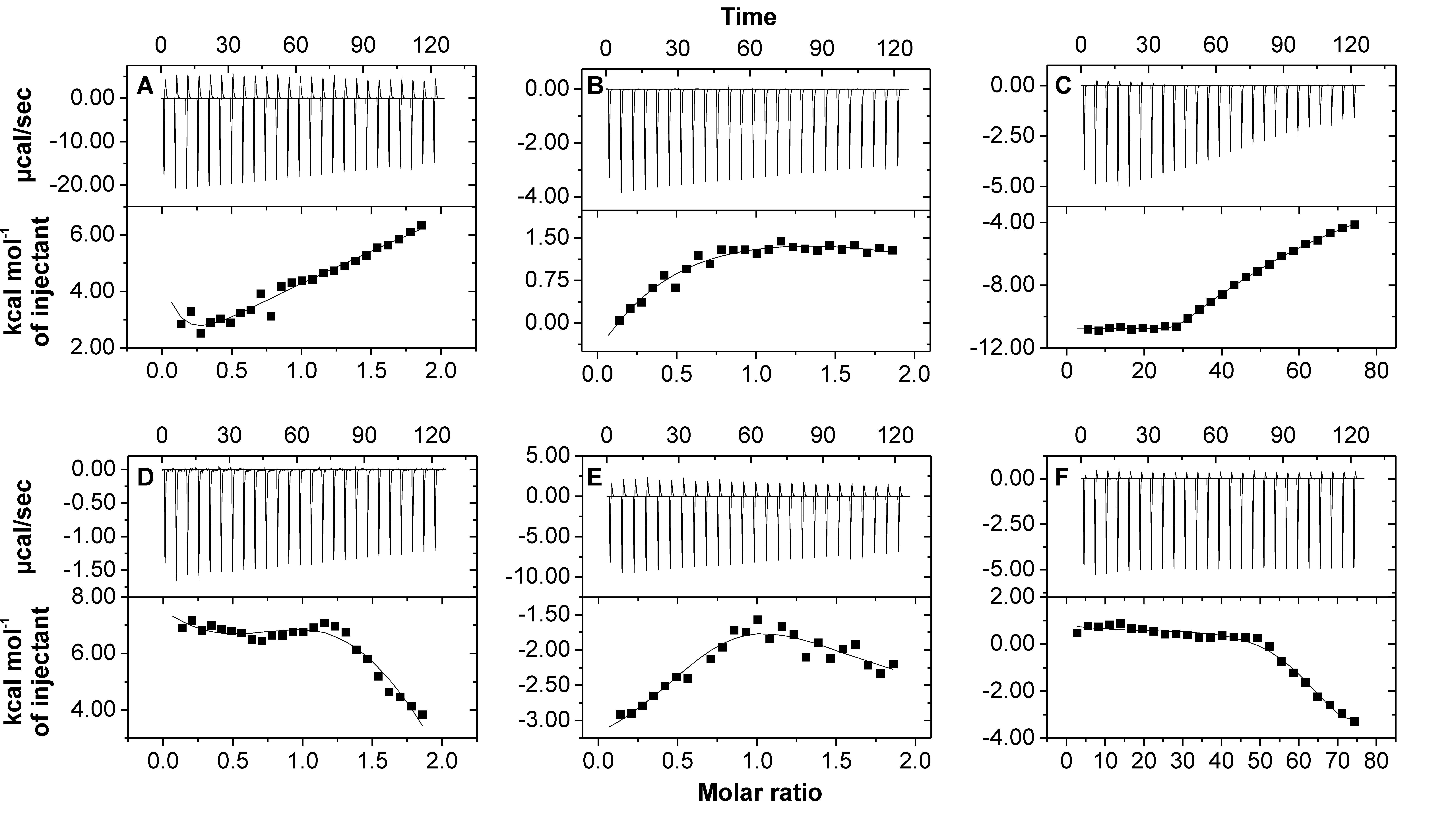


**Figure S2:** ITC results showing the heat released during the titration of cysteine (100.0 µM) by **1** (1.0 mM; **A**) or *cisplatin* (1.0 mM; **D**), GSH (100.0 µM) by **1** (1.0 mM; **B**) or *cisplatin* (1.0 mM; **E**), and HSA (2.5 µM) by **1** (1.0 mM; **C**) or *cisplatin* (1.0 mM; **F**) at 30 °C in the water/DMF mixture (1:1 v/v), and the binding isotherms with the heat of dilution from the blank experiment subtracted. The data were fitted to a two-site (for **B** and **C**), three-site (for **A**, **E** and **F**) or four-site (for **D**) binding models (solid lines).





**Figure S3:** Effects of the tested complexes and the reference drug *cisplatin* on MMPs secretion in isolated tumors. Tumors from mice without any treatment are labeled as Control. The results are presented as mean±S.E. # significant difference in comparison to *cisplatin*-treated cells (*p <* 0.05).


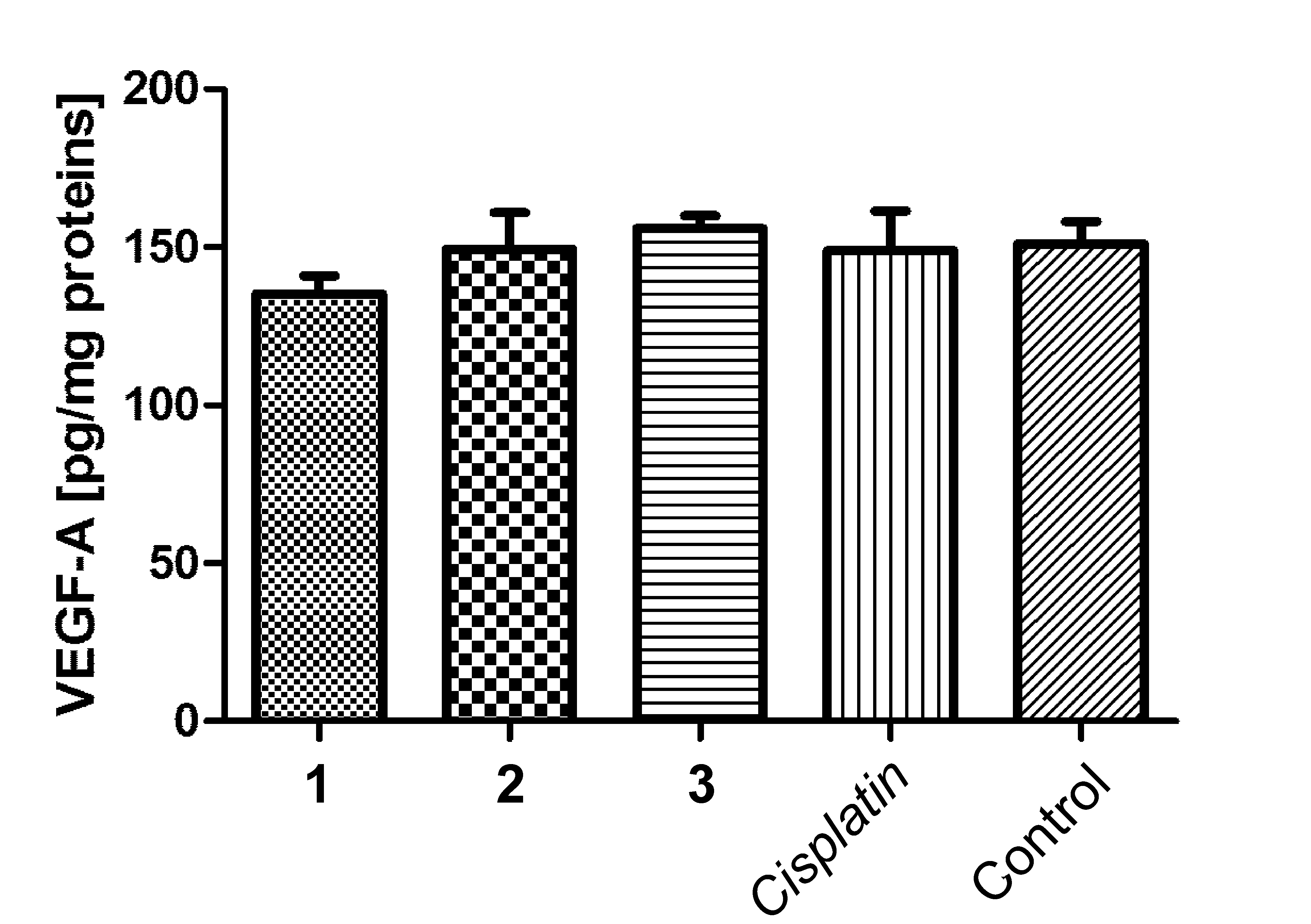


**Figure S4:** Effects of the tested complexes and the reference drug *cisplatin* on VEGF-A secretion in isolated tumors. Tumors from mice without any treatment are labeled as Control. The results are presented as mean±S.E.

**Table S1**

*In vitro* antitumor activity of the complexes **1**–**3** and *cisplatin* given as logIC_50_ (μM).

| Cell line | **1** | **2** | **3** | *Cisplatin* | mean |
| --- | --- | --- | --- | --- | --- |
| A549 | 0.86 | 1.01 | 0.69 | 1.41 | 1.08 |
| HeLa | 0.65 | 0.70 | 0.63 | 1.00 | 0.77 |
| G-361 | 0.30 | 0.48 | -0.22 | 0.53 | 0.35 |
| A2780 | 0.41 | 0.38 | 0.26 | 1.08 | 0.67 |
| A2780R | 0.43 | 0.45 | 0.32 | 1.43 | 0.94 |
| MCF7 | 0.53 | 0.90 | 0.30 | 1.29 | 0.92 |
| HOS | 0.58 | 0.59 | 0.40 | 1.53 | 1.04 |
| LNCaP | 0.52 | 0.58 | 0.18 | 0.58 | 0.49 |
| mean | 0.57 | 0.69 | 0.39 | 1.23 |  |
